# Utilization of the Primary Health Care Services in the Tshwane Region of Gauteng Province, South Africa

**DOI:** 10.1371/journal.pone.0013909

**Published:** 2010-11-09

**Authors:** Thembi P. Nteta, Matilda Mokgatle-Nthabu, Oluwafemi O. Oguntibeju

**Affiliations:** 1 School of Public Health, MEDUNSA, University of Limpopo, Pretoria, South Africa; 2 Department of Biomedical Sciences, Faculty of Health and Wellness Sciences, Cape Peninsula University of Technology, Bellville, South Africa; Kenya Medical Research Institute, Kenya

## Abstract

**Background:**

In South Africa, the provision of primary health care is a basic service designed to be cost effective and bring healthcare as close as possible to the population, particularly to those people of low economic status. It is a service which is provided free of charge by the South African government and as part of the millennium health goals, it is intended that the service should be accessible to the populace and be effectively utilized.

**Objective:**

This study was designed to investigate the accessibility and utilization of the primary health care services in three community health care centres in the Tshwane of the Gauteng Province, South Africa.

**Methodology:**

Data were obtained from participants attending three Community Health Care Centres in the Tshwane Region using self-administered structured questionnaires. A document review of the Community Health Care Centres records was conducted to investigate the utilization trends of the services provided and descriptive statistics were used to analyze the data obtained.

**Results:**

The results showed that the Community Health Care Centres in the Tshwane Region are accessible to most participants who lived within 5 km of such centres and who traveled 30 minutes or less to the clinic. Using a taxi or walking were found to be the most common means of transport used to gain access to such a clinic. The findings showed that generally, participants were satisfied with the services provided.

**Conclusion:**

Participants of this study have access to the community health care centres in the Tshwane Region and there seems to be effective utilization by patients attending them.

## Introduction

Primary Health Care (PHC) was adopted as a basic mechanism to promote health care to the population of South Africa and is delivered through the District Health System [1]–[3]. The South African health system adopted the PHC approach because it was thought to be the most acceptable and cost effective means of improving the health status of her citizens in particular the less privileged. This system brings healthcare as close as possible to where people live and work and it constitutes the first element of a continuing health care process. It was formally introduced in South Africa in April 1994 and the South African government is the main service provider of PHC [4].

In 1994, two policies were implemented, namely ‘Free health for pregnant mothers and children under the age of 6 years’ and ‘Universal access to PHC for all South Africans.’ The emphasis was on the improvement and development of clinics and essential health care programmes, like mother and child health care, nutrition, expanded immunization and management of communicable diseases and the introduction of the termination of pregnancy (Act No 92 of 1996) which gave a legal right to women on reproductive choices [1], [5], [6].

A comprehensive PHC service package was developed in September 2001 by the South African Department of Health. The purpose of this package was to outline comprehensive PHC services, which within a period of 5 years of implementation, should be available to the population throughout the country. It is believed that an integrated package of essential PHC services should be made available to the entire population and should also provide a solid foundation for a single unified health system. It was anticipated that this package would contribute to greater social needs and promote equity by reducing the gap between those who have access to an appropriate level of healthcare and those who do not [4], [7].

It also provides guidance for provincial and district health authorities on how to optimally provide these services and formulate plans to bring services acceptable to national standards. The overall objective was to improve access to high quality and effective healthcare and reduce inequalities between the PHC services. The services to be provided include: immunization, mother and child care services, antenatal and postnatal care including family planning, sexually transmitted disease care, treatment of minor ailments and curative services, mental health, school health, treatment of chronic diseases (e.g hypertension and diabetes), treatment of communicable diseases (e.g tuberculosis and HIV/AIDS), oral health, rehabilitative services also and the provision of essential drugs. Clinics should also render a comprehensive integrated PHC service using a one-stop approach for at least eight hours a day, five days a week [4], [7].

It has been reported that over 400 clinics in South Africa have been constructed and upgraded. To an extent, the PHC services have helped to improve the utilization rate of the primary health care services as communities now travel shorter distances to health care centres, however there is still shortage of equipment, staff and essential drugs [7].

In 2003, Gauteng Province had 86% of facilities that were open to the public for five days or more per week, with a small percentage (5%) of the primary health care services providing 24-hour services. This is due to the distribution of the population, being clustered in big cities where hospitals provide emergency services for a number of neighbouring clinics [2]. Despite positive reports on PHC services, there is still a significant number of people in South Africa who do not have adequate access to health care services due to geographical, physical, population growth, language and other barriers [1], [6], [7].

Tanser, Gijsbertsen, Berbst [8] reported that geographical accessibility of health services has a direct bearing on the utilization of such services. Distance to a facility was reported to be associated with increasing maternal and infant mortality, decreased vaccination coverage and decreased contraceptive use. Improving geographical access to a PHC can help to improve these adverse health outcomes.

According to Tlebere *et al.* [9], transportation and distance from a PHC facility were the biggest hindrances to the utilization of health services, particularly in rural areas. A lack of financial resources for transport was the barrier most often cited by women who did not attend antenatal clinics. Some other barriers include limited financial resources, the influence of family members, family responsibilities, women not realizing they are pregnant and experiencing difficulty in obtaining time off from work to attend a clinic.

It has been shown that anti-retroviral services in South Africa is not being effectively utilized because of stigma; health workers negative behavior and the problem that people are required to frequently travel to a health facility to collect their medication [10].

The purpose of this study therefore was to assess the accessibility and utilization of the Community Health Centres (CHCs) in the Tshwane Region, Gauteng Province, South Africa.

### Statement of the problem

A study by Rispel *et al*. [11] indicated that Primary Health Care services in South Africa are experiencing overcrowding, long queues of people, long waiting times and a lack of resources. Despite the benefits of adopting and implementing the district healthcare system, health care services have remained inaccessible in many respects and have therefore negatively affected its optimum utilization. Because of the problems associated with accessibility and utilization of primary health care services, this study was designed to identify shortcomings in the delivery of primary health care services in the Tshwane region. It is hoped that the findings of this study will provide insights into the problems of accessibility and utilization and help to improve primary health care service delivery.

## Methods

### Study design

A cross-sectional, descriptive survey study design was employed in which self-administered structured questionnaires were used to assess accessibility of services. A document review of community health centres' records were also used to investigate the utilization trends of the services provided.

### Study setting

The study was conducted at three Community Health Centres (CHCs) in the Tshwane Region. All the 3 CHC's which are provincial clinics were selected to avoid bias. They all provide PHC services over a 24 hour period, thus they were purposively selected. These three CHCs (A, B and C) are:

- CHC A situated in the northern part of Tshwane with an estimated population of 310 000, mainly Blacks of low to middle socio-economic status.
- CHC B situated in the central part of Tshwane with an estimated population of 256 000, mainly Blacks of low to middle socio-economic status.
- CHC C located in the southern part of Tshwane with an estimated population of 20 000, mainly Indians of middle to high socio-economic status [12].

### Sampling and sampling method

A simple random sampling technique was used for data collection. In this technique, all persons attending the clinic had an equal chance of being selected for the study. Using the register of attendees who registered to utilize the clinic for each day, 100 participants were sampled at each CHC and the sample size was calculated according to the method of White [13].

Ninety-six (33.7%) of participants were above 45 years of age, followed by 62 (21.8%) with an age range of 26 to 35 years; parents who brought their children (<5 years to the clinic were 50 (17.5%) (Note: parents/guardians responded on behalf of the under aged children), 42 (14.7%) ranged from ages 16 to 25 years and 35 (12.3%) ranged from 36 to 45 years with the average mean age being 18.5 years.

Of the 300 questionnaires distributed, two hundred and eighty-five questionnaires were correctly filled in, giving a response rate of 95%.

### Data collection and outcome measures

A self-administered structured questionnaire adapted from [6] was used to obtain quantitative data from the participants. Participants were interviewed face-to-face by clinic staff and interviewers were trained for three days using standard manual as well as role-plays. Questionnaires were translated from English language to Setswana (the local language) and back translated using a standard WHO protocol. The quality of translations was independently verified by bilingual experts before distribution of questionnaires to participants.

The questionnaires were divided into two sections i.e a demographic and a clinic information section. The questionnaires were given randomly to people who utilized the clinic on a specific day. The CHCs on average examined 500 people per day and 100 people (20%) were assessed using structured questionnaires according to the method of Brink [13]. The study was carried out over an eight month period (01 October 2007-31 May 2008).

The questionnaire assessed (outcome measures) staff component, utilization trends, age (total number of people, number of patients seen by a nurse per day, number of patients seen by doctor per day, total number of patients seen at the CHC for that month, total number of patients who utilized the service, curative service (child health and minor ailments), sexually transmitted diseases, immunization, preventive service (antenatal care, family planning and Pap smear), HIV (VCT and ARV), chronic diseases (hypertension, diabetes and asthma), referrals (to the doctor, hospital or specialized services) and pharmacy (items being used). The register or computerized/electronic monthly statistical records were examined using a data extraction method to obtain data covering the period from January 2005 to December 2007.

### Data analysis

Descriptive statistics were used to analyze the data. The analysis was based on completed questionnaires and extracted data from records at the three community health centres. In analyzing the data, an assessment of the monthly data on clinic attendance and utilization was conducted. Data were imported into SSPS software. Analysis included frequency distributions of different types of services utilized and the results are presented by making use of bar charts and tables. Statistical significance was set at P<0.05.

### Reliability and validity

A pilot study was done to test the questionnaire. By doing a pilot study the feasibility of the study was investigated (the validity of the measuring tools and the acceptability of the study to the study population) so that potential problems could be identified and resolved before commencing the study. The information gained was used to improve the methods/instrument where applicable. The pilot study was conducted on a small group of people (ten) at one of the CHCs. The findings of the pilot study assisted the investigators in the removal of questions that were considered to be vague or unclear to the participants.

### Ethical considerations

Approval to perform the study was obtained from the School of Public Health, Research, Ethics and Publications Committee (REPC) and Medical Research and Ethics Committee (MREC), University of Limpopo, South Africa.

Informed consent was obtained from study participants. Participants were given information to enable them to make decisions to either participate in the study or not. Parents/guardians gave consent for their children who were under 18 years and responded to questionnaire on behalf of their children. The benefit of taking part in the study was explained to the participants (to improve the quality of health care services). Participants were assured that their identity would remain anonymous and that all the discussions held would be confidential.. Consent forms and questionnaires were in both English and Setswana in order to address any language barriers.

Permission to use clinics and clinics' records was obtained from the Department of Health and all data obtained from the clinic records were kept confidential and were used solely for the purpose of the study.

## Results

### Demographic information

#### Participants

A total of 285 participants completed the questionnaire. A large proportion of the participants were self respondents (235), of which 75 (26.33%) were males and 160 (56.2%) were females. Parents/guardians who responded on behalf of their children were 50 (17.5%), of which 15 (5.3%) were male and 35 (12.2%) female.

#### Gender

**Figure 1:**
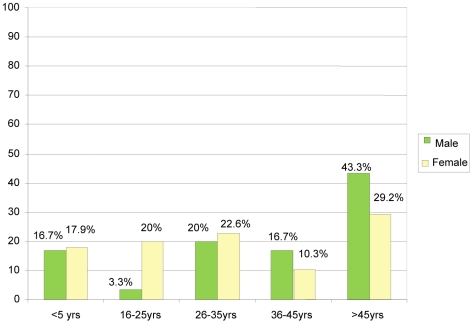
Showed the distribution of the participants by gender with male (green colour) and female (yellow colour) represented in bars and highlights the percentage of male and female participants according to their age group.

There were more female (195) than male participants (90). Among the females, those above 45 years were 57 (29.2%), followed by 44 (22.6%) from 26 to 35 years of age, 39 (20%) from 16 to 25 years and 20 (10.3%) were between 36 to 45 years of age. Among the males, 39 (43.3%) were above 45 years of age, followed by 18 (20%) with an age range of 26 to 35 years and 15 (16.7) were 36 to 45 years old and 3 (3.3%) were 16 to 25 years of age (Figure 1).

#### Age

Ninety-six (33.7%) of participants were above 45 years of age, followed by 62 (21.8%) with an age range of 26 to 35 years; parents who brought their children (<5 years old children) to the health centres and responded on their behalf were 50 (17.5%), 42 (14.7%) ranged from ages 16 to 25 years and 35 (12.3%) ranged from 36 to 45 years with the average mean age of 18.5 years.

#### South African citizenship

The respondents were asked to indicate whether they were South African citizens or not.

Of the 285 participants, 274 (96%) said they were South African citizens. Of these, 189 (66%) were females and 85 (29.8%) were males. Only 11 (3.9%) said they were not South African citizens (6 females and 5 males).

#### Distance traveled

**Figure 2:**
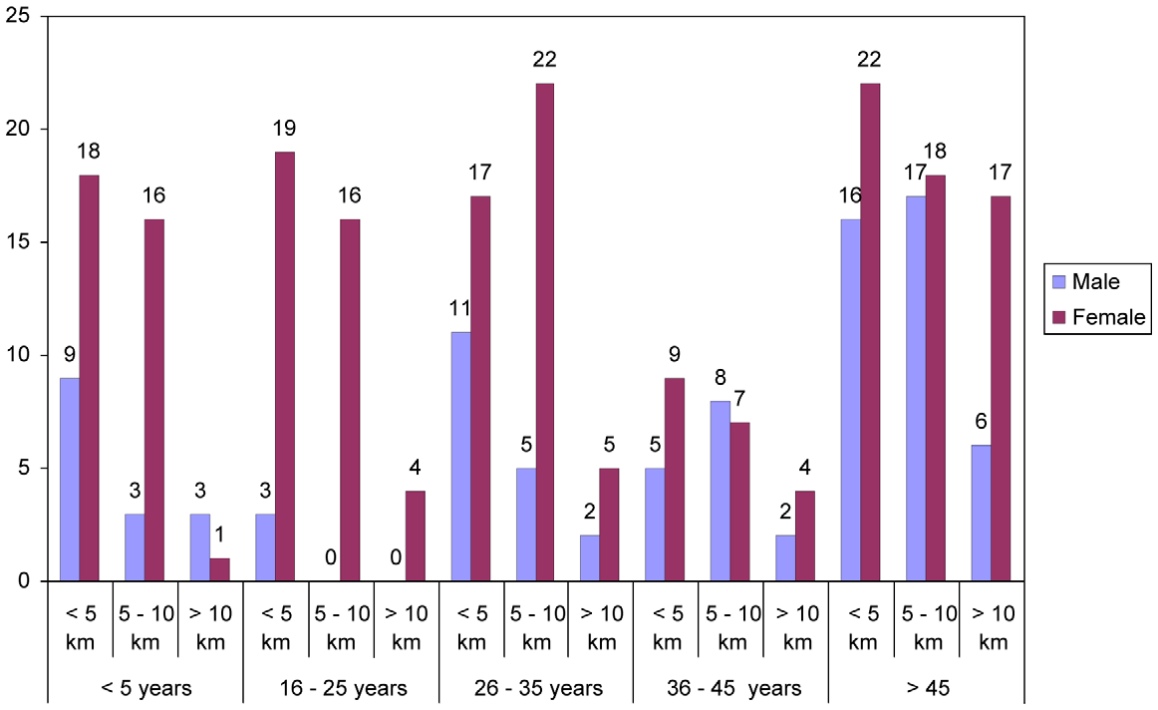
Shows the distance covered by the participants in terms of gender which is represented in bars (male-purple colour and female-wine colour). The age of the participants ranged from <5 years to >45 years.

The distance travelled by the respondents to the clinic was one of the parameters used to assess accessibility as indicated by Figure 2.

Figure 2 shows that a significant proportion (P<0.05) of the participants 129 (45.3%) traveled less than 5 km to the clinic, of which 67 were females, 35 males and 27 children. Participants who reported to have traveled 5 to 10 km were 112 (39.2%), of which 63 were females, 30 males and 19 children. Those who traveled more than 10 km were 44 (15.4%), of which 30 were females and 10 males and 4 children under the care of the parents.

#### Travelling time

**Figure 3:**
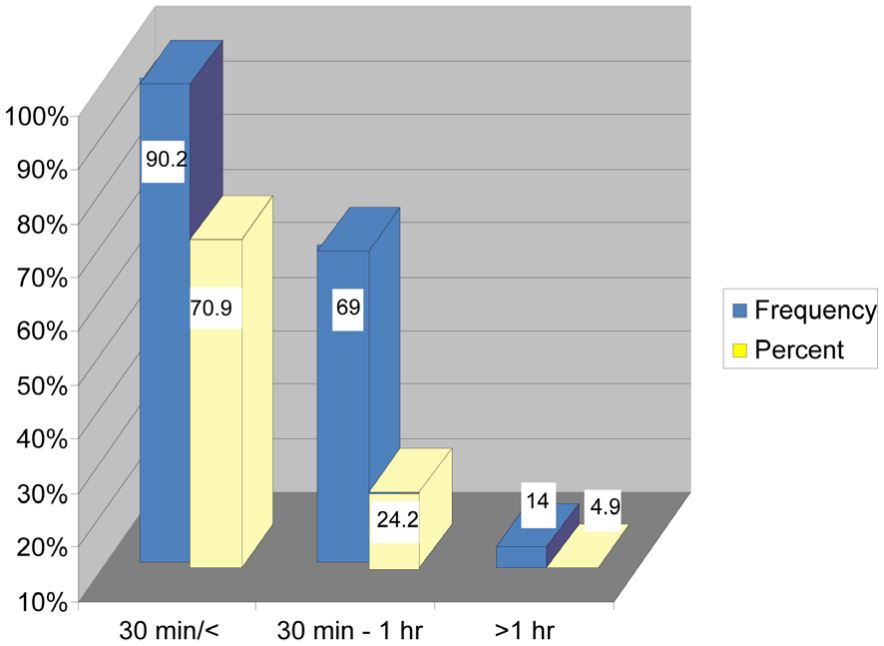
shows distance covered in minutes/hours by the participants. The minutes/hours covered by the participants are represented on the X-axis while the percentage is represented on the Y-axis. The frequency and percentage of distance covered by the participants is represented by blue and yellow colour respectively.

Figure 3 shows that 202 (70.9%) of the respondents reported to have traveled for 30 minutes or less to the clinic. Those who traveled for 30 minutes–1 hour were 69 (24.2%) and those who traveled longer than an hour were 14 (5%). There was a significance difference (P<0.05) between participants who spent 30 minutes to 1 hour to access health care facility and those who spent 1 hour or longer than 1 hour.

#### Means of transport

Participants were asked to specify the means of transport used to reach the clinic. Of the participants 134 (47%) used a taxi as a mode of transportation to travel to the clinic. The second most common mode of transport was walking 123 (43.2%). Twenty-six (9.1%) used their own transport and two people used a bus.

#### Reasons for coming to the clinic

The respondents were asked to indicate the reason(s) for visiting the clinic and also to indicate their different disease conditions (Table 1).

**Table 1:** Distribution of reasons for coming to the clinic.

Age (Years)	Diabetes		Hypertension		Tuberculosis		Asthma	
	Male	Female	Male	Female	Male	Female	Male	Female
<5 yrs	0	0	0	0	0	0	0	1
16–25 yrs	1	0	1	2	0	0	0	0
26–35 yrs	1	5	1	2	3	1	2	3
36–45 yrs	5	2	4	5	4	7	2	3
> 45 yrs	23	22	2	3	21	36	4	3
**Sub Total**	30	29	8	12	28	44	8	10
**Total**	59		20		72		18	

#### Diabetes

The number of participants who utilized the clinic due to diabetes were 59. Males above 45 years were 23 (76.7%), followed by 5 (16.6%) within the age range of 36 to 45 years, and 1 (3.3%) each for age 26 to 35 years and 16 to 25 years. Females above the age of 45 were 22 (75.9%), 5 (17.4%) for age 26 to 35 years, and 2 (6.9%) for age 36 to 45 years.

#### Hypertension

Respondents who utilized the health care service due to hypertension were 20 (12 females and 8 males). Four males (50%) ranged from 36 to 45 years, 2 (25%) above 45 years, and 1 (12.5%) in each between 16 to 25 years and 26 to 35 years. Females between the ages of 36 to 45 years were 5 (41.7%), 3 (25%) were above 45 years, followed by 2 (16.7%) each with their age ranging from 16 to 25 and 26 to 35 years respectively.

#### Tuberculosis

Of the seventy-two respondents who reported to attend the clinic due to tuberculosis, 44 were females and 28 males. 36 (81.8%) females were above 45 years (highest number), 7 (16%) were within the age range of 36 to 45 years, followed by 1 (2.3%) from 26 to 35 years, while males above 45 years were 21 (75%). Four respondents (14.3%) were in the age range of 36 to 45 years, and 3 (10.7%) were from 26 to 35 years.

#### Asthma

Those who reported to attend the clinic due to asthma were 18 (10 females and 8 males). There were 4 (50%) males above 45 years, and 2 (25%) respondents in the age range of 26 to 35 years and 36 to 45 years respectively. There were 3 (30%) females above 45 years and those in the age range of 26 to 35 and 36 to 45 comprised 3 (30%). There was 1 (10%) child below the age of 5 years brought in by their parents.

#### Pap smear

Of the 285 participants, only 20(7%) females were reported to have visited the clinic for a Pap smear.

#### Antenatal care

Results showed that a total of 34 (11.9%) females out of the 285 participants utilized the clinic for antenatal care (pregnancy check-up). The other 251 (88.1%) participants came for various reasons.

#### Voluntary counseling and testing (VCT)

Table 2 shows the number, age group and gender of respondents that came to the clinic for voluntary counseling and testing. Documentation records of the clinic showed that 39 respondents came to the clinic for HIV Voluntary Counseling and Testing (23 females and 15 males).

**Table 2:** Distribution of Age, Gender and VCT.

Age (Years)	VCT	
	Male	Female
16–25 yrs	1	12
26–35 yrs	8	8
36–45 yrs	5	2
> 45 yrs	2	1
**Sub Total**	16	23
**Total**	39	

#### Antiretroviral treatment

The number of respondents, age group and gender that reported to the clinic for anti-retroviral treatment is shown on the Table 3.

**Table 3:** Age Gender ARV Cross tabulation.

AGE (Years)	ARV	
	Male	Female
16–25 yrs	0	2
26–35 yrs	5	6
36–45 yrs	1	1
> 45 yrs	3	4
**Sub Total**	9	13
**Total**	22	

Table 3 shows that twenty-two respondents reported to the clinic for anti-retroviral treatment (13 females and 9 males).

### Clinic utilization

#### Distribution of clinic utilization

Of the 285 participants, 209 (73%) people were reported to have utilized just one of the different services available while seventy-six (27%) people utilized 2 to 3 of the services.

#### Doctor or nurse

It was reported that 77 (80.2%) participants above 45 years of age were seen by a nurse and 19 (19.8%) people were seen by a doctor. Reports showed that all the 50 (100%) children below 5 years old were seen by a nurse. In the age group of 26 to 35 years, 48 (77.4%) were seen by a nurse and 14 (22.4%) by a doctor. Thirty-seven (88.1%) of those within the age range of 16 to 25 years were seen by a nurse and 5 (11.9%) by a doctor. Of the age range of 36 to 45 years, 32 (91.4%) were seen by a nurse and 3 (8.6%) by a doctor.

#### Referral

Of 285 participants, 232 (81%) were not referred to other services. Those referred to the doctor were 35 (12%) while those who were referred to the hospital were 13 (5%). It was reported that only 5 (2%) were referred to specialized services.

#### Prescription and medicine issued

Respondents were asked about their prescriptions and medicine issued according to their age (Table 4).

**Table 4:** Distribution of prescription and medicine issued against age.

Prescription			
Age	Yes	No	Total
<5 yrs	5	45	50
16–25 yrs	23	19	42
26–35 yrs	45	17	62
36–45 yrs	29	6	35
>45 yrs	93	3	96
Total	195	90	285

Medicine Issued			
Age	Yes	No	Total
<5 yrs	5	0	5
16–25 yrs	22	1	23
26–35 yrs	45	0	45
36–45 yrs	27	2	29
>45 yrs	91	2	93
Total	190	5	195

#### Prescription issued

A total of 195 prescriptions were issued.

#### Medicine issued

Of the 195 prescriptions that were issued, 190 people received their medicine and only 5 people did not receive medication.

#### Health needs

A large proportion of the participants 271 (95%) stated that their health needs or requests were met. However, 14 participants' (5%) indicated that their health needs or requests were not met.

#### Service availability

There was a significant difference (P<0.05) in service availability and non-availability. The majority of the participants (264) reported that services were available. However, 21 reported that services were not available.

#### Satisfaction with service and time

Two hundred and thirty-six participants were satisfied with the services and the opening time's available whilst 49 participants were not satisfied. Our results showed that a significant number (P<0.05) of the participants were satisfied with the services rendered at the Community Health Centres.

#### Recommendation and change

Out of 285 participants, 258 said that they would recommend a friend or family to come to the clinic when sick. The other 27 participants would not recommend a friend or family to come to the clinic, and 190 respondents were of the opinion that nothing requires any attention or needs to be changed at the clinic. The other 95 participants felt that change in the management of the clinic is required.

## Discussion

### Demographic data

#### Age

The majority of participants were females at the time of the study. The services most probably attracted females due to the kind of services being offered as antenatal care, family planning; Pap smear and immunization are more female-orientated. There were no specialized male services, such as urology being offered.

#### Accessibility: Distance, travelling time and means of transport

Access to a primary health care facility is seen as a basic social right [14]. For effective utilization of health care services, access is important and it is seen as a major health and development factor. Interestingly, most governments declare that their citizens should enjoy equitable access to primary health care services but disparities have been shown to exist between the poor and the rich with respect to access to health care services. Although, it is difficult to define and measure access, however, health service studies commonly define access as utilization rates. In our study majority of the participants indicated that they had access to the primary health care which significantly helped in the utilization rates of these services.

It is known that that dissatisfaction with primary care services especially in the public sector leads many individuals to health care shop [15] or to visit higher level hospitals for primary care which consequently lead to considerable inefficiency and loss of control over efficacy and quality of services. In developing countries such as South Africa, the effect of distance on service use becomes stronger when combined with the dearth of transportation and poor roads which contributes towards increase costs of visits [16]. Availability of transport, physical distance of the facility and time taken to reach the facility has been shown to influence health service utilization. It has also been reported that the distance separating patients and clients from the nearest health facility has been remarked as an important barrier to use primary or community health care facilities, especially in rural areas [17].

The current study showed that in terms of distance, the clinics were accessible as most of the participants lived within 5 km of such a facility. According to the set norms and standards of the South African Primary Health Care Services, access is measured by the proportion of people living within 5 km of a clinic [4].

Similarly, a study done by Bell *et al.* [18] in Namibia concluded that accessibility was said to be satisfactory as people in urban Windhoek traveled short distances to the clinic. Chatora and Tumusime [19] pointed out that in sub-Saharan Africa, health centres were set up so that people in the catchment area would not travel more than 8 km or spend more than one hour travel time to access the nearest health facility.

A significant proportion of participants reported to have traveled 30 minutes or less to the clinic. Others traveled between 30 minutes to an hour and only a minority traveled for more than an hour (Figure 3). The travelling time in Tshwane, Gauteng Province is reported to be shorter when compared to that of Hlabisa Health Sub-district, Kwa Zulu Natal Province, South Africa, where it has been reported that the median travel time to the nearest clinic was 81 minutes, and that 65% of the people traveled for 1 hour or more to attend the nearest clinic [8].

The results indicated that the most common form of transport used by the respondents to access health care services was a taxi, secondly by walking and only a few people used their own transport. Tanser, Gijsbertsen, Herbst [3] reported that in accessing health care facilities at Hlabisa health sub-district, Kwa Zulu Natal, South Africa, people often walked, which was followed by public transport. Some individuals reported that they had to walk very long distances because there was no public transport available in their area or they were too poor to afford the fare.

In 1998, it was estimated in South Africa that nationally 49%, 41% and 10% of black South Africans walked, used public transport and their own transport respectively, to access health care [20]. In the current study, the rates for Tshwane Region were 43.2%, 47% and 9.1% respectively. The higher use of own transport at the expense of walking in the national survey probably reflects the urban influence. Public transport usage and walking were the most popular mode of transport to access the health services. These results are in agreement with the study by Tanser, Gijsbertsen, Herbst [3] that has shown a relationship between proximity to a clinic and the proportion of the population choosing to walk to the clinic.

### Clinic information and utilization of services at the three CHCs in the Tshwane Region

Generally, under-utilization of health services in public sector is viewed as a universal phenomenon in developing countries and that client-perceived quality of services and confidence in the health provider affect health service utilization. The type of illness or symptoms experienced for the particular illness and duration are all known to affect health service utilization [21]. The results of this study revealed that the Tuberculosis (TB) clinic was the most frequently visited service. It has been reported that South Africa has the fifth largest number of people living with TB in the world [4]. The South African national treatment success rate of 51% is below the World Health Organization's target of 85%. In 2005, there were 320, 000 cases of TB recorded in the country. The reported TB cases have almost doubled in Gauteng province since 1997. It increased from 241 per 100 000 in 1997 to 379 per 100 000 in 2007. TB has been further complicated by co-infection with HIV [4]. TB is treatable but requires a combination of drugs that are taken over several months. Due to this extended treatment period, many people do not complete their treatment and are therefore not cured. In 1996, in recognition of this problem, the Minister of Health declared TB a health priority and thus marked the introduction of Directly Observed Treatment, Short Course (DOTS) as a national strategy to address the problem [4]. Because of the directly observed treatment by health workers, it may explain the reason why the TB clinic is mostly visited also as reported in this study.

The survey revealed that all the services provided at the clinics were effectively utilized. Our findings agree with that of Pillay [7] who reported an increased utilization of primary health care services in KwaZulu-Natal Province. Also, an earlier survey by the South African Health Systems Trust and Department of Health 2003 showed that the utilization of services in Gauteng Province has increased although it differs according to the type of services offered [22].

The current study showed that the majority of the participants were satisfied with the service and hours of operation. However, a minority of the participants stated that they were not satisfied because of the long queues of people at the CHCs and they sometimes prefer to go home without getting the appropriate service. Also, that diagnostic procedure, such as X-rays were not always available due to for an example X-rays machine being “forever broken down”. A few respondents reported that in one or two centres a dentist was not present for most of the time and that staff shortages; slow service delivery and negative attitude of health care staff prevailed.

Rispel *et al*. [11] also identified health care system barriers that included long waiting periods, unfriendly and uncaring behaviour of health workers as well as poor health facilities. Despite those health care barriers, a significant number of participants had access to the health care facilities and were satisfied with available services and said they would recommend to a friend or family to utilize the clinic when sick.

### Limitation of the study

Due to time and financial constraints, it was impossible to include all the clinics in the Tshwane Region for the purpose of this study. There are 28 local authority clinics and 9 provincial clinics in the region. The investigators therefore conducted the study at the three community health centres that operate a 24-hour service and provided all the primary health care services. Therefore, the results cannot be generalized to all clinics that are only open for 8 hours per day in Tshwane. Neither can the findings be generalized to health services in other provinces or in the whole country.

### Recommendations

Based on the findings of the current research, the following recommendations are made which could be implemented to further improve the accessibility and utilization of Primary Health Care Services:

- The Department of Health should increase the number of community health centres in the Tshwane region and also increase the number of health centres offering 24-hour services which render all primary health services.
- More doctors and especially nurses should be appointed at the existing primary health centres since most of the patients are seen by nurses.
- Working conditions for staff should be improved by improving the health workers salaries and encouraging professional development, i.e. to train more nurses as primary health care practitioners and specialize in their field of interest. This would improve the quality of care.
- The Department of Health should develop and implement an Employee Assistance Programme to support health care personnel, which might help to eliminate the unfriendly and uncaring behaviour of health care personnel.
- The government should subsidize services, such as a bus service to and from the clinic, plus laboratory tests since everybody has a right to health.

### Conclusion

According to the Constitution of South Africa, 1996, everyone has the right to have access to health care services. The primary health care approach was adopted in order to promote accessibility to the use of health services. Overall, participants indicated that they had access to the primary health care services in Tshwane and were satisfied with the services provided.
